# A Lifetime Achievement in Bioengineering: Professor Shu Chien

**DOI:** 10.1007/s10439-019-02390-2

**Published:** 2019-10-24

**Authors:** Sheldon Weinbaum, Yi-Shuan Julie Li, Geert W. Schmid-Schönbein

**Affiliations:** 1 Department of Biomedical Engineering, The City College of New York, New York, NY 10038 USA; 2 Department of Bioengineering, University of California San Diego, 9500 Gilman Dr., La Jolla, CA 92093-0412 USA

**Figure:**
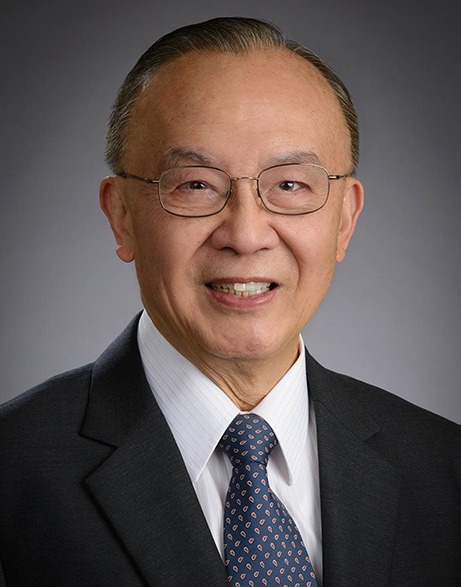
Shu Chien

Any historian who looks at the development of Bioengineering as an emerging engineering discipline in the twentieth and twenty-first Century, will recognize Shu Chien as one of the cornerstones and foremost matchmakers who facilitated the marriage between engineering and medicine. Being trained in Medical School in Taiwan, with a PhD Degree in cardiovascular physiology at Columbia University under the mentorship of Magnus I. Gregerson, Shu Chien exhibited an extraordinary ability to pursue interdisciplinary research opportunities for engineering. At a time when the engineering approach to most medical problems was unexplored, Shu’s inspiring enthusiasm combined with his superb diplomat skills served to build the bridges that allowed the growth of a new field of engineering in medicine. Shu naturally surrounded himself with engineering and life sciences investigators and facilitated their effective collaboration long before this practice was established. He was a key protagonist in creating bioengineering as a new discipline.

Chien’s early work focused on rheological properties of blood. He was the founding director of the Laboratory for Hemorheology at Columbia University, where he developed a major interest in the measurement of the biophysical properties, and especially the non-Newtonian and viscoelastic properties of blood. His team carried out some of the most detailed measurements of apparent viscosity and analyzed the mechanisms for blood’s non-Newtonian properties due to red cell deformability and aggregation.^5^ During a sabbatical at the University of Gothenburg with Professor P.-I. Brånemark, Shu was introduced to Professor Richard Skalak, Professor of Mechanical Engineering and later the Director of the Bioengineering Institute at Columbia University. They saw in Brånemark’s laboratory the remarkable deformation of red cells in human capillaries and recognized the need to develop a description of the biophysical properties of the red cell membrane. They started a collaboration and soon were surrounded by an enthusiastic group of biomedical engineers that tested the mechanical properties of single cells with tools like micropipettes. Shu directed the systematic application of engineering methods with well-defined mechanical stresses in combination with measurements of the red cell membrane shape. They lead eventually to the first constitutive law for red cell membranes deformation with Richard Skalak.^19^ This constitutive law has become the standard in cell membrane mechanics and served to predict all types of red cell behavior, like the parachute shape in single cell file capillaries, in passing through narrow pores, deformation in a red cell rouleaux, during membrane tank treading and other applications. The work also served to establish a viscoelastic model of the red cell membrane^3^ and was the basis for refinements that allow more and more detailed engineering analysis of motions and interactions of red blood cells.

As a medical doctor, Chien focused on the importance of applying this newly gained insight to analyze blood circulation in patients. He provided fundamental assessments of rheological properties of blood in sickle cell patients^4^ and other diseases.

As the understanding of red cell rheology was progressing, Shu Chien’s team added leukocytes to the analysis of blood rheology. This pioneering step brought to light the differences in the biophysical properties of circulating leukocytes and red cells and opened new ways of looking at leukocytes in circulation, especially microcirculation. Measurements of the comparatively large size, unique morphology and high stiffness of leukocytes^16^ compared to red cells provided the background for discovery of unique hydrodynamic interactions between red cell and leucocytes in single file capillaries. They are the precondition for allowing leukocytes to start rolling on the endothelium as well as for their adhesion to its membrane.^17^ The team also discovered the surprising spontaneous activation of circulating leukocytes in patients that leads to the entrapment of leukocytes in microvessels and even obstruction of capillaries. The work sparked a long-term search for the origin of spontaneous leukocyte activation in patients by Schmid-Schönbein.

Shu saw the need to establish a research direction which looked at blood rheology entirely in the living circulation. With the onset of techniques to measure pressure, flow rates, hematocrit in vessels of the microcirculation he embarked with Herbert Lipowsky and his long-term colleague, Shunichi Usami, on a program to study blood rheology in living blood vessels.^14^ These studies brought to light that basic principles in blood rheology established by in vitro studies are also seen in vivo, but that there are also features unique to the in vivo environment not reproduced by in vitro studies, especially in pathophysiological conditions. The first measurements of apparent viscosity in vessels of the microcirculation down to the size of individual capillaries were made.

Initiated through a fortunate meeting with Shelly Weinbaum of The City College of New York, facilitated by YC Fung in 1969, Shu developed an interest in endothelial transport aspects of arterial disease. In the early 1970’s researchers became interested in the fluid dynamic aspects of arterial disease and in particular the role of fluid shear stress in endothelial permeability. The initial discussion was whether early lesions formed in the high or low shear regions of the major arteries. Permeability was measured using fluorescently labeled tracers and it was clearly demonstrated that low shear regions had higher permeability. The central mystery was how a molecule as large as LDL (22 nm) could cross the endothelial lining when there were no obvious pores for a molecule of this size since the clefts between the endothelial cells (ECs) were significantly smaller even where there were breaks in the tight junction strands that held the cells together. Initially it was widely believed that the pore was open vesicles which decorated the luminal surface. As proposed by Professor George Palade, then at Yale University, the vesicles could detach and migrate by Brownian motion to the opposite membrane of the endothelial cells and thus serve a ferry boat function. In 1984 Shu Chien and Anne Baldwin reported critical experiments with cationized ferritin which suggested that this transendothelial transport does not occur.^2^ Decades later the vesicles attached to the endothelial cell membrane were shown to serve as fluid shear-stress shelters for membrane receptors.^18^

In 1985 the Chien–Weinbaum team proposed a new paradigm for transendothelial LDL transport, namely that the leakage was caused by endothelial cell turnover during the death and sloughing off of dying cells and replacement by neighboring healthy cells during cell division, mitosis.^23^ This was hypothesized to be a rare occurrence and thus very difficult to observe. Low shear regions would have greater cell death than high shear regions with unidirectional flow. A theoretical model was developed to predict the quantitative feasibility of the concept and this model led to an NSF “Special Creativity Award” to design and perform a validating experiment. By observing the entire surface of a rat aorta with 500,000 endothelial cells one was able to show that only one cell in several thousand on average was undergoing cell turnover at a given time and that this rate of turnover differed in low and high fluid shear regions around flow branches.^13^

As a follow up to these experiments, Chien and Weinbaum developed both a theoretical model and experiments to examine at the cellular level the growth of LDL leakage spots surrounding cells in turnover, the spread of tracer in the arterial intima around these leakage sites and the role of fenestral pores in the underlying internal elastic lamina.^10^ David Rumschitzki, and Kung Ming Jan were also key contributors. Chien and Weinbaum continued their collaboration for nearly 30 years. Their final paper^25^ explored the rolling of leukocytes on the endothelial glycocalyx and the penetration of leukocyte microvilli in the rolling process.

In the late 1970s Shu saw the next major opportunity to deepen the understanding of the cardiovascular system by incorporating molecular biology into engineering analysis of living systems. He committed to the integrated research of the biochemical and mechanical regulations in cardiovascular systems in health and diseases. The line of research systematically investigated the molecules, signaling, the processes of transcription and translation, epigenetics regulations under the influence of mechanical stresses encountered in the circulation. It forecast the advancement of vascular cell biology, tissue engineering, and systems biology. The approaches contributed to the advancement of mechanobiology, physiology and tissue engineering.

In 1988, after 31 years as a Professor at Columbia, Shu moved with Richard Skalak, Shunichi Usami, Paul and Amy Sung to the University of California San Diego UCSD and joined B.W. Zweifach, Y.C. Fung, and M. Intaglietta, who had established a bioengineering academic program. In 31 years at UCSD, Shu led his team and worked with collaborators globally to study the hemodynamic regulation of vascular biology. He systematically investigated the cellular events in biochemical and mechanical environments.

The focal nature of the arterial lesion demonstrates the impact of mechanical environment on the pathophysiological process in the arterial tree. The lesion prone area possesses the disturbed blood flow pattern, whereas the lesion protective area experiences a steady laminar flow. As already recognized, the lifetime of endothelial cells on the inner lining of blood vessels depends greatly on their local flow environment. Shu’s studies focused on how they respond to different blood flow patterns to modulate their gene expression, phenotypical changes, as well as their communications with other vascular cells, which in turn lead to the modulation of vascular homeostasis.^6^ Shu investigated the mechanosensing mechanisms (including cell surface integrins, receptor tyrosine kinases, G protein receptors, ion channels, as well as cell membrane structures),^12^ cell mechanics (including the dynamics of focal adhesion sites, cellular junction, and cytoskeleton),^26^ mechanotransduction cascades (including Rho, AMPK, PI3 K, NFkB, and MAKP, pathways), transcription machinery (including transcription factors),^22^ and regulation (including mRNAs, microRNAs, and long-non-coding RNAs),^1^,^15^,^20^ as well as epigenetic regulations (including DNA and histone modifications).^8^,^27^ Shu not only mastered the conventional methodologies for comprehensive studies, but also pioneered the state-of-art technologies to investigate the mechanophysiology in the cardiovascular system. With collaborations, Shu employed the fluorescence resonance energy transfer {FRET} technology to construct biosensors monitoring cellular events with high temporal and spatial resolution to elucidate the sequences and prorogation of mechanotransduction in single living cells in real time.^21^

Shu was not only a leading contributor to the advancement of current knowledge about cellular mechanotransduction and regulation in the cardiovascular system, he also was a leader in the development of novel tools for treatment of disease. With colleagues, Shu developed a platelet-coated-nanoparticle technology to carry the target to the injured site in the vessels for high efficacy treatment. Furthermore, Shu extended the knowledge gained in cardiovascular studies to multidisciplinary fields, with the best example being stem cell research. With his colleagues he developed the platform to investigate the effects of different microenvironments (including substrate composition, rigidity, and topology) on stem cell renewal and differentiation.^7^,^9^,^24^

His rich and pioneering record of scientific achievements is summarized in more than 617 peer reviewed scientific reports, a small selection of which is listed below. The names of his many collaborators are in these reports and in a Festschrift on the occasion of his 80^th^ Birthday.^11^ Shu received more than 30 research awards, many from professional engineering societies including the Pierre Galletti Award from the American Institute of Medical and Biological Engineering (AIMBE) in 2004 and two Melville Medals from the ASME, the only person with this distinction. Shu was elected to the National Academy of Engineering in 1997 and received their Founders Award in 2006. In 2005 he became a member of all three US National Academies, NAE, NAS and NAM at a time when there were only six other living individuals with this honor. Among many other honors, he received in 2012 the National Medal of Science by President Obama. Besides his scientific contributions he made major contributions to the developing infrastructure of bioengineering in the USA and world-wide, serving as Founding Chair of the Department of Bioengineering at UCSD, the UCSD Institute of Engineering in Medicine, and the California Institute for Bioengineering. He was President of BMES and of AIMBE, facilitating the creation of the National Institute of Biomedical Imaging and Bioengineering which was signed into law by President Clinton on December 29, 2000.

Professor Shu Chien will retire in October 2019. On behalf of colleagues and friends around the world we salute him with the best wishes for the next stage of his life. We thank him for his leadership and a life-time of friendship.
